# Awareness of and willingness to use PrEP among Black and Latinx adolescents residing in higher prevalence areas in the United States

**DOI:** 10.1371/journal.pone.0234821

**Published:** 2020-07-06

**Authors:** Tamara Taggart, Yilin Liang, Paulo Pina, Tashuna Albritton

**Affiliations:** 1 Department of Prevention and Community Health, George Washington University, Milken Institute School of Public Health, Washington, DC, United States of America; 2 Department of Social and Behavioral Sciences, Yale School of Public Health, New Haven, Connecticut, United States of America; 3 Loyola University Chicago Stritch School of Medicine, Maywood, Illinois, United States of America; 4 Department of Pediatrics, New York University (NYU), Brooklyn, New York, United States of America; 5 Department of Community Health and Social Medicine, City University of New York (CUNY) School of Medicine, New York, New York, United States of America; University of North Carolina at Chapel Hill, UNITED STATES

## Abstract

This mixed-methods study examined awareness of and willingness to use pre-exposure prophylaxis (PrEP) among sexually active Black and Latinx adolescents (13–17 years) residing in five cities in the United States with some of the highest burden of HIV. Data are from adolescents who participated in a cross-sectional survey (n = 208) and one-on-one interviews and focus groups (n = 26) conducted from September 2017—August 2019. Approximately 50% of the sample were recruited through community efforts, and the other half through a panel. Logistic regression with covariates including sexual orientation, relationship status, socioeconomic status, and race/ethnicity were used to assess factors associated with PrEP awareness and willingness. For the qualitative data, thematic analysis was used to develop a codebook of a-priori and inductive codes while analytic memos were written to identify key themes. PrEP awareness was reported by 38% of the sample and was associated with Black race (AOR = 0.49; 95% CI = 0.27, 0.90) and prior HIV testing (AOR = 3.89, 95% CI = 1.25, 12.08). PrEP willingness (defined as “definitely would use PrEP”) was reported by 22% of the sample and was associated with higher age, more education, having had condomless sex in the past 6 months (AOR = 0.23; 95% CI = 0.10, 0.56), perceived likelihood of acquiring HIV (AOR = 3.59; 95% CI = 1.06, 12.21), and PrEP awareness (AOR = 0.41; 95% CI = 0.19, 0.89). Qualitative data showed that misconceptions about PrEP persist and PrEP stigma, fear of being punished, provider attitudes and recommendations, and empowerment were related to adolescents’ willingness to use PrEP. Study findings reveal important strategies for improving PrEP delivery and scale-up to Black and Latinx adolescents. These strategies include using sociodemographic and health behavior data to target adolescents who may be more or less willing to use PrEP, improving provider communication about PrEP, and creating culturally and developmentally appropriate PrEP education materials that address common misconceptions held by adolescents.

## Introduction

Although the HIV/AIDS epidemic is stabilizing for many groups, adolescents (for the purposes of this study, we define adolescents as people aged 13 to 17 years), young men who have sex with men (MSM), and racial/ethnic minority populations continue to have significant HIV risk. In the United States (U.S.), adolescents and young adults aged 13 to 24 years account for an estimated 21% of all new HIV diagnoses [1]. The majority of these infections occur among Black and Latinx youth [1]. Adolescents have a greater risk of contracting HIV due to many contextual, social, and biological factors including cognitive functioning, identity formation, experimentation, and increased autonomy [2, 3]. Despite advances in HIV prevention and treatment, few youth access the biomedical treatments available to prevent transmission of HIV [1, 4].

Oral antiretroviral pre-exposure prophylaxis (PrEP) is recognized as a promising biomedical prevention strategy for people at risk for HIV [5]. In 2018, the U.S. Federal Drug Administration (FDA) approved daily PrEP for HIV prevention for adolescents at risk for HIV. Studies on the safety and feasibility of PrEP showed that despite challenges in adherence, PrEP is well tolerated by adolescents [6]. However, PrEP uptake among adolescents in general, and racial/ethnic minority adolescents in particular, has been slow due to lack of awareness of PrEP, behavioral factors, and legal and financial barriers to adolescents seeking sexual and reproductive health care [7–9]. Increasing PrEP uptake among adolescents at risk for HIV has the potential to reduce new infections and address persistent profound racial disparities in HIV [10, 11]. Although a number of studies examine PrEP awareness and interest among young adults [12–14], limited attention has been given to sexually active racial/ethnic minority adolescents.

Given that HIV continues to disproportionately affect Black and Latinx adolescents, investigations of the factors associated with their potential PrEP use are needed. Understanding these factors will inform developing strategies to target adolescents with biomedical HIV prevention. The current study was conducted to examine awareness of and willingness to use PrEP among a sample of sexually active at-risk Black and Latinx adolescents in five urban areas in the U.S. that have been most affected by HIV [15].

## Materials and methods

Data are from the Who’s on Board study, a community-based mixed-methods study conducted from September 2017 –August 2019. During the study, 208 adolescents completed a survey. Approximately 50% (n = 109) of the sample were recruited through community efforts (flyers in youth servicing organizations and community health clinics, direct recruitment in adolescent primary care clinics and emergency departments, and pediatric physician referral) in New York, NY and Washington, DC; the others (n = 99) were recruited through a Qualtrics panel distributed to adolescents residing in Newark, NJ; Philadelphia, PA; and Baltimore, MD. Additionally, we recruited n = 26 adolescents to participate in focus groups and one-on-one interviews. Focus group and interview participants were recruited from youth servicing organizations and community health clinics in New York, NY.

### Study population

Adolescents were eligible to participate in the study based on the following criteria: (1) self-identified as Black and/or Latinx/Hispanic, (2) between the ages of 13 and 17 years, (3) self-identified as HIV negative, (4) English fluency, and (5) sexually active (defined as having had anal and/or vaginal intercourse at least once in the past 12 months).

### Procedures

We used a convergent parallel design to simultaneously collect qualitative and quantitative data [16, 17]. Surveys were used to assess overall trends and to identify factors associated with adolescent PrEP knowledge and attitudes; focus groups and interviews were conducted to provide insight into why certain beliefs and attitudes around PrEP exist and to corroborate findings from the surveys. Adolescent survey participants provided informed assent and completed a survey using a self-administered questionnaire on Qualtrics, either online using a personal electronic device (e.g., smartphone) or in a designated research space at a health clinic using a tablet (direct recruitment). Survey assessments were anonymous and took approximately 35 minutes to complete. Participants received a $20 gift card for completing the survey and were directed to a community resource page with information on PrEP and adolescent sexual health. Adolescent focus group and interview participants provided informed assent and parental consent. Focus groups were approximately 70 minutes and one-on-one interviews ranged from 28 to 38 minutes with an average time of 34 minutes. Cis-gender women and men participated in three focus groups separated by gender, while four Black and Latinx MSM participated in one-on-one interviews. At the recommendation of our community partners, MSM (i.e., men who identified as gay or bisexual) were asked to participate in a one-on-one interview while heterosexual men and heterosexual, lesbian, and bisexual women participated in focus groups. Focus groups and interviews were conducted by either a Black woman or a Latinx man trained in interviewing techniques with racial/ethnic and sexual minority adolescents. Focus group and interview participants received a $25 gift card for their participation. The survey and semi-structured qualitative guides were developed by a team of adolescent and young adult community members, PrEP clinicians, and investigators with expertise in adolescent sexual health. The survey and qualitative guides were pre-tested with Black and Latinx adolescents (n = 5) prior to data collection and refined to increase readability, understanding, and relevance. All study procedures and instruments were approved by the Institutional Review Boards at Yale University and St. Barnabas Hospital System.

### Measures

Behavioral and sociodemographic items were assessed using questions from the Youth Risk Behavior Surveillance system. Specifically, survey participants were asked to report the number of sexual partners in the past six months, condom use in the past six months, drug and alcohol use during sex in the past six months, and whether or not sexual partners were known to be HIV positive. We also asked participants if they had ever been tested for HIV, tested for sexually transmitted infections (STI), and if they had ever had a positive diagnosis of an STI (including chlamydia, gonorrhea, or syphilis). We assessed perceived likelihood of becoming HIV-infected through the question, “What do you think the chances are that you will ever get HIV in your lifetime?” Possible response options included “not at all likely”, “a little bit likely”, “somewhat likely”, “very likely”, and “extremely likely”. Perceived likelihood of becoming HIV positive was dichotomized as “not at all likely or a little bit likely” and “extremely/ very/or somewhat likely”.

HIV knowledge was assessed using an 12 item scale [18, 19]. Sample items included: “Birth control pills can protect a woman from sexually transmitted infections including HIV”, “Vaseline and other oils should not be used to lubricate condoms”, and “Most people who have an STI or HIV look and feel healthy”. Response options included “definitely false”, “probably false”, “don’t know”, “probably true”, and “definitely true”. HIV knowledge was scored such that a definitely correct answer received a “1” and an incorrect answer received a “0”.

PrEP awareness was assessed with the question “Have you ever heard of a daily pill that an HIV-negative person can take to prevent getting HIV” and the response options were “yes”, “no”, or “I don’t know” [20]. Prior to completing the other PrEP measures in the survey, adolescents were provided with a brief summary of PrEP including background information, availability, dosing, costs, and side effects. Willingness to use PrEP was assessed by asking participants “How likely would you be to take PrEP if it were available for free?”. Response options were, “definitely would not take PrEP”, “probably would not take PrEP”, “might take PrEP”, “probably would take PrEP”, and “definitely would take PrEP”. Willingness to use PrEP was defined as a participant indicating “definitely would take PrEP”.

Participants were also asked to self-report their sociodemographic characteristics. The following items were assessed: race/ethnicity, age (years), current grade/level of education (year), gender (i.e., female, male, transgender woman, transgender man, gender queer, other), sexual orientation (i.e., asexual, bisexual, gay, heterosexual/straight, lesbian, queer, other, prefer not to say), and relationship status (i.e., in a relationship, not in a relationship). Socioeconomic status was assessed by asking youth “In general, would you say you and your family have more money than you need, just enough for your needs, or not enough to meet your needs.”

A semi-structured focus group and interview guide assessed adolescents’ knowledge and attitudes about HIV prevention, PrEP, sexual and social norms, and barriers and facilitators to accessing HIV prevention. Prior to discussing PrEP, participants were provided with a brief summary of PrEP. The following questions were used to elicit responses about adolescents’ awareness of and willingness to use PrEP for HIV prevention: (a) What have you heard about PrEP or the “HIV prevention pill”?; (b) What are some of the concerns people your age might have about getting a prescription for PrEP from a doctor?; (c) What are the chances that adolescents prescribed PrEP would take it daily?; (d) Would you tell your parents/friends/boyfriend/girlfriend or sexual partner that you are taking PrEP?; and (e) How do you think your parents/friends/boyfriend/girlfriend or sexual partner would feel if you told them you wanted to take PrEP?. Focus group and interview participants also completed a brief sociodemographic questionnaire.

### Data analysis

We analyzed the quantitative and qualitative data separately and integrated results in the overall interpretation phase of the study [16, 17]. For survey participants, sample demographics and descriptive statistics were examined. Bivariate analyses were conducted using chi-square and t-test to evaluate independent associations between PrEP awareness and sociodemographics, HIV testing, STI diagnosis, and other HIV-related variables. Factors with a statistically significant association (*p*< 0.10) were selected and further explored using a multivariable logistic regression model. The final adjusted models included items that were significant (*p*< 0.05) and variables that are believed to be confounders based on findings from our bivariate analyses and a review of literature these included sexual orientation, relationship status, socioeconomic status, and race/ethnicity. The same procedures were followed to assess willingness to use PrEP. Participants recruited from community efforts or the panel were not statistically different in awareness of PrEP or willingness to use PrEP, and therefore we did not control for this factor in logistic regression models. All quantitative analyses were conducted using SAS version 9.4.

All focus groups and interviews were digitally recorded, transcribed verbatim, and edited to remove identifiers. After multiple readings by the research team, transcripts were uploaded to Dedoose, a qualitative data management and analysis software. The research team used thematic analysis [21] to develop a codebook of a-priori and inductive codes. Analytical memos were written throughout to identify key themes and further refine codes. We discussed any differences in coding until we reached agreement. Qualitative data were grouped into themes to explicate upon quantitative findings.

## Results

### Participant sociodemographic characteristics

The survey sample consisted of 208 adolescents. About half of the sample identified as female (53%) (see Table 1 for sample characteristics). A little over half of the sample were Latinx (59%). The mean age was 15.9 years (± 1.11). Approximately 58% of the sample were heterosexual, 13% had 3 or more sexual partners in past 6 months, 44% reported being in a relationship, 44% of the sample reported having had condomless sex in the past 6 months, and 43% of the sample reported being under the influence of alcohol or drugs during their last anal or vaginal sexual intercourse. Of those who had ever been tested for an STI, 13% reported being diagnosed with an STI. HIV knowledge score was an average of 6.33 (± 5.66) out of a possible 12. The qualitative sample consisted of 26 adolescents (see Table 2 for sample characteristics). About half of the sample identified as female (54%). Approximately 42% identified as Black/African American and approximately 12% had 3 or more sexual partners in the past six months.

**Table 1:** Descriptive statistics for the survey sample (N = 208).

	All survey participants (N = 208)	%
Age, years (Mean ± SD)	15.92 (1.11)	
Education, years (Mean ± SD)	10.45 (1.29)	
Race/Ethnicity		
Black/African American	86	41.4
Hispanic/Latinx	122	58.7
Socioeconomic Status		
More than needed	19	9.1
Just enough	137	65.9
Not enough	52	25.0
Gender		
Female	111	53.4
Male	90	43.3
Transgender woman	0	0
Transgender man	1	0.5
Gender queer	5	2.4
Other	1	0.5
Current Relationship Status		
Yes	91	43.8
No	104	50.0
Don’t know	13	6.3
Sexual Orientation		
Lesbian	2	1.0
Gay	4	1.9
Bisexual	32	15.4
Heterosexual	120	57.7
Queer	6	2.9
Asexual	3	1.4
Other/ Prefer not to say	41	19.7
Number of Sexual Partners, past 6 months		
≤ 3	181	87.0
> 3	27	13.0
HIV Test		
Yes	96	46.2
No/don’t know	112	53.8
Ever Diagnosed with STI		
Yes	27	13.0
No	181	87.0
Condomless Sex, past 6 months		
Yes	92	44.2
No	99	47.6
Had ever heard of PrEP		
Yes	79	38.0
No/don’t know	129	62.0
HIV Knowledge (Mean ± SD)	6.33 (5.66)	

**Table 2:** Descriptive statistics for the focus group and interview sample (N = 26).

	All focus group and interview participants (N = 26)	%
Age, years (Mean ± SD)	15.32 (1.22)	
Race/Ethnicity		
Black/African American	11	42.3
Hispanic/Latinx	9	34.6
Multi-racial	6	23.1
Socioeconomic Status		
More than needed	4	15.4
Just enough	18	69.2
Not enough	4	15.4
Gender		
Female	14	54.0
Male	12	46.0
Sexual Orientation		
Lesbian	1	3.8
Gay	1	3.8
Bisexual	7	27.0
Heterosexual	15	57.7
Queer	2	7.7
Number of Sexual Partners, past 6 months		
≤ 3	21	80.8
> 3	3	11.5

### PrEP awareness: “Isn’t that like the HIV medication?”

Approximately 38% of the survey sample were aware of PrEP. Adjusted and unadjusted odds ratios for factors associated with PrEP awareness are presented in Table 3. Compared to Black/African American participants, Latinx participants had a lower odds of being aware of PrEP (AOR = 0.49; 95% CI = 0.27, 0.90). Those who had ever received HIV testing had higher odds of being aware of PrEP compared to participants who had never received HIV testing (AOR = 3.89, 95% CI = 1.25, 12.08). Focus group and interview data support these quantitative results and provide further insight into factors that influence PrEP awareness. Most qualitative participants were unaware of PrEP or had misconceptions about PrEP and its use. For example, a focus group participant shared, “I know that it’s something that people use—men, specifically—use to prevent HIV and AIDS.” Others in this focus group agreed and shared that they saw advertisements on social media or heard about PrEP from popular television shows as a way to prevent HIV in men. Participants in the one-on-one interviews described PrEP as a medication “you take before you have HIV” and that they heard about PrEP at their local health clinic and also saw advertisements on social media. Despite these descriptions, most participants reported that they had never heard of PrEP or were unsure about its use.

**Table 3:** Adjusted and unadjusted odds ratios (and 95% confidence interval) for factors associated with PrEP awareness among participants (N = 208).

	Unadjusted OR (95% CI)	p-value	Adjusted OR (95% CI)	p-value^a^
Age, years (mean)	1.09 (0.64, 1.85)	0.86		
Education, years (mean)	0.90 (0.57, 1.41)	0.70		
Race/Ethnicity				
Black/African American	1.00		1.00	
Hispanic/Latinx	0.36 (0.17, 0.74)	0.006	0.49 (0.27, 0.90)	**0.02**
Gender				
Female	1.00			
Male	0.75 (0.38, 1.49)	0.95		
Transgender man	<0.001 (<0.00, >999.99)	0.98		
Gender queer	<0.001 (<0.00, >999.99)	0.96		
Other				
Sexual Orientation				
Lesbian	<0.001 (<0.00, >999.99)	0.96		
Gay	>999.99 (<0.00, >999.99)	0.89		
Bisexual	1.32 (0.43, 4.05)	0.98		
Heterosexual	1.00			
Queer	<0.001 (<0.00, >999.99)	0.93		
Asexual	1.13 (0.09, 14.67)	0.98		
Other/ Prefer not to say	0.59 (0.25, 1.42)	0.99		
Current Relationship Status				
Yes	1.79 (0.86, 3.75)	0.17		
No	1.00			
Don’t know	0.69 (0.12, 4.10)	0.59		
HIV Test				
Yes	1.47 (0.72, 3.03)	0.31	3.89 (1.25, 12.08)	**0.02**
No/don’t know	1.00		1.00	
Condomless Sex, past 6 months				
Yes	1.00			
No	0.74 (0.37, 1.47)	0.35		
Likelihood of Acquiring HIV				
Extremely/Very/Somewhat Likely	3.96 (1.04, 15.05)	0.04		
A little bit/Not at all Likely	1.00			

^a^Statistically significant results (*p*< 0.05) are highlighted in bold

### PrEP willingness: “I feel like some people are in denial about the disease…because it’s like everywhere. Also, it’s embarrassing sometimes for some people”

**Fig 1:**
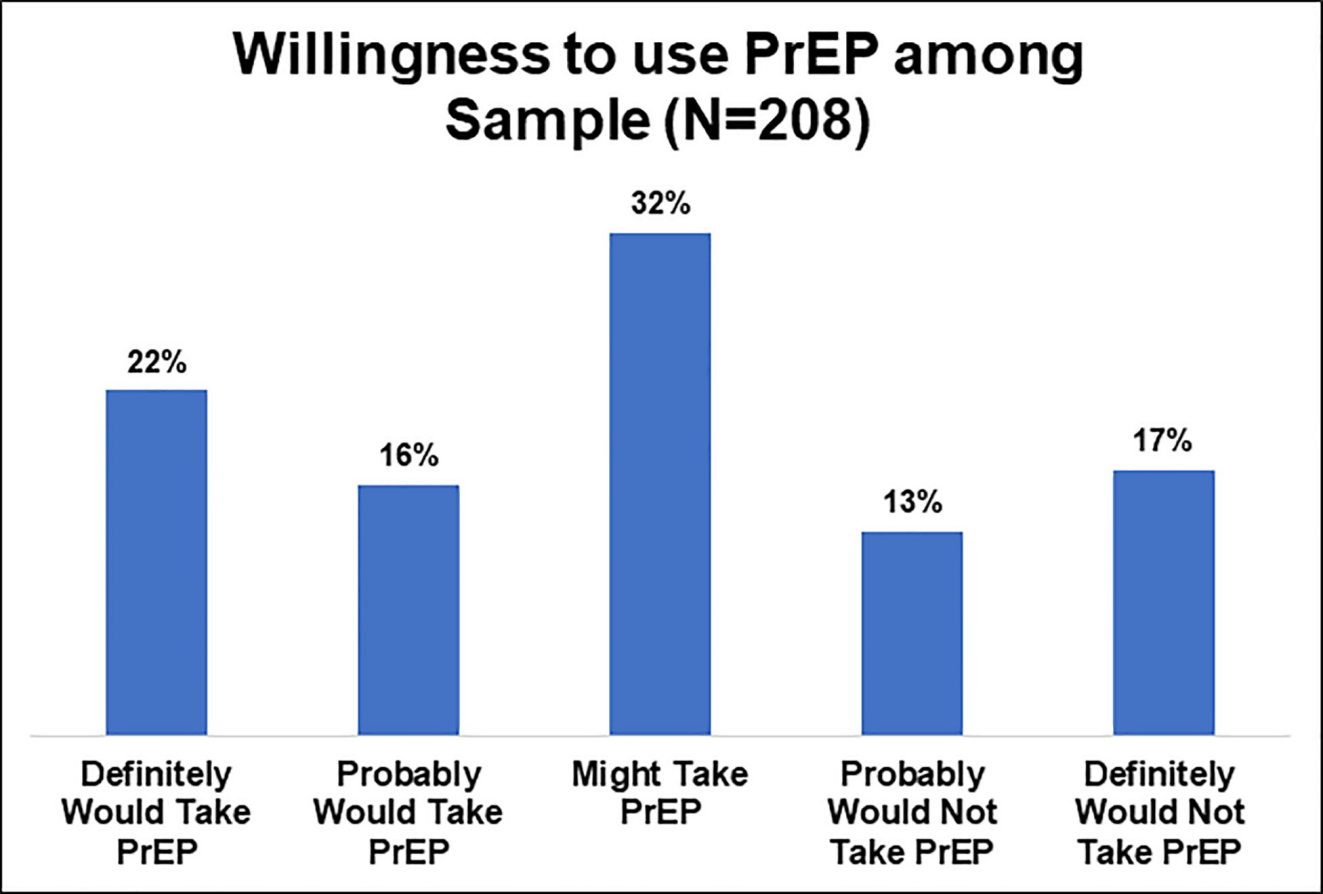
Willingness to use PrEP among survey sample (N = 208).

Adjusted and unadjusted odds ratios for factors associated with willingness to use PrEP are presented in Table 4. Approximately 22% of the sample were willing to use PrEP (Fig 1). In the adjusted model, factors associated with interest in PrEP were age, education, condomless sex, perceived likelihood of acquiring HIV, and PrEP awareness. Higher age and more education were significantly associated with willingness to use PrEP. Participants who did not have condomless sex in the past 6 months were less likely to be interested in PrEP compared to participants who had condomless sex in the past 6 months (AOR = 0.23; 95% CI = 0.10, 0.56). Participants who reported being extremely/very/somewhat likely of acquiring HIV had higher odds of being willing to use PrEP compared to participants who reported a little bit/not at all likely of acquiring HIV (AOR = 3.59; 95% CI = 1.06, 12.21). Those who had not heard of PrEP were less likely to be willing to use PrEP compared to those who had heard of PrEP (AOR = 0.41; 95% CI = 0.19, 0.89).

**Table 4:** Adjusted and unadjusted odds ratios (and 95% confidence interval) for factors associated with willingness to use PrEP among participants (N = 208).

	Unadjusted OR (95% CI)	p-value	Adjusted OR (95% CI)	p-value^a^
Age, years (mean)	0.43 (0.21, 0.89)	0.02	0.43 (0.22, 0.84)	**0.01**
Education, years (mean)	2.13 (1.13, 4.02)	0.02	1.97 (1.11, 3.49)	**0.02**
Race/Ethnicity				
Black/African American	1.00			
Hispanic/Latinx	0.50 (0.20, 1.23)	0.129		
Gender				
Female	1.00			
Male	0.74 (0.32, 1.74)	0.98		
Transgender man	<0.001 (<0.00, >999.99)	0.97		
Gender queer	5.93 (0.28, 127.28)	0.95		
Other				
Sexual Orientation				
Lesbian	<0.001 (<0.00, >999.99)	0.96		
Gay	2.87 (0.20, 41.17)	0.87		
Bisexual	0.79 (0.21, 2.97)	0.90		
Heterosexual	1.00			
Queer	<0.001 (<0.00, >999.99)	0.95		
Asexual	<0.001 (<0.00, >999.99)	0.96		
Other/ Prefer not to say	0.88 (0.30, 2.54)	0.90		
Current Relationship Status				
Yes	0.77 (0.30, 2.01)	0.25		
No	1.00			
Don’t know	2.34 (0.38, 14.43)	0.28		
HIV Test				
Yes	0.88 (0.36, 2.15)	0.77		
No/don’t know	1.00			
Condomless Sex, past 6 months				
Yes	1.00		1.00	
No	0.24 (0.09, 0.61)	0.003	0.23 (0.10, 0.56)	**0.001**
Likelihood of Acquiring HIV				
Extremely/Very/Somewhat Likely	3.98 (0.97, 16.32)	0.06	3.59 (1.06, 12.21)	**0.04**
A little bit/Not at all Likely	1.00		1.00	
Had ever heard of PrEP				
Yes	1.00		1.00	
No/I don’t know	0.50 (0.21, 1.16)	0.10	0.41 (0.19, 0.89)	**0.02**

^a^Statistically significant results (*p*< 0.05) are highlighted in bold

Turning to our qualitative findings, adolescents provided a number of reasons for why they would be more or less interested in PrEP if it were available for free. Most who were unwilling to use PrEP expressed concerns about being judged by their parents or other family members for needing PrEP. For example, focus group participants shared that people their age are unwilling to use PrEP because, “they’re probably afraid that their parents are going to find out”. Other participants shared similar fears of being punished or judged by their family stating, “Yeah, I’m doing that [taking PrEP] and they [other family members] gonna look at me differently”. Other reasons for being unwilling to take PrEP included concerns about side-effects and “being consistent” with a daily pill. Participants also discussed the role of health care providers in their willingness or unwillingness to take PrEP. Those who expressed discomfort with their provider and felt that their provider “would look at me like, you’re a teen and you’re asking for these pills, like what are you doing” were unwilling to take PrEP. Further describing that their fear of being seen as promiscuous or “bad” by their provider would make them unwilling to take PrEP. By contrast, some participants shared that they would be more willing to take PrEP if a provider recommended it to them because they trust their provider. One participant shared, “I think that the doctor should be like, I recommended that you take this daily pill because it can help lower the chance of you getting HIV”. Lastly, empowerment and the ability to protect oneself were other reasons adolescents were willing to take PrEP. One focus group participant shared that people their age would be willing to take PrEP “because now, I have a way to protect myself over this thing that killed [a family member]”. Adolescents also shared that telling their sexual partners about PrEP would increase their willingness to use it because they would “just feel more happy. Like, yeah, he’s protected”.

## Discussion

This mixed-methods study is among the first in the U.S. to document awareness of and willingness to use PrEP among Black and Latinx adolescents residing in cities that are most affected by HIV [15]. Introduction to and engagement in PrEP is needed during adolescence to curb the growing spread of HIV among Black and Latinx youth. While a number of studies investigate factors associated with PrEP awareness and interest in young adults, limited attention has been given to sexually active racial/ethnic minority adolescents.

PrEP awareness among Black and Latinx adolescents in this study is similar to other studies with community samples of Black and Latinx young adults [12, 22, 23]. Our finding that PrEP awareness is associated with prior HIV testing may be related to higher risk perception and greater exposure to PrEP promotion messages for adolescents who have received an HIV test. This finding supports using HIV testing as an entry point for biomedical and behavioral HIV prevention, including PrEP [24]. Further, the lower frequency of testing within the sample and its association with PrEP awareness highlights the need to continue to increase HIV testing among Black and Latinx adolescents. Our finding that Latinx adolescents have a lower odds of PrEP awareness suggests the need to develop strategies that increase access to developmentally and culturally congruent PrEP information for Latinx adolescents. This finding also affirms the importance of meaningfully engaging Latinx youth in developing interventions that address cultural and normative barriers to PrEP [25, 26].

Most adolescents in our sample are unwilling to use PrEP if it was made available to them for free. There are two plausible explanations for our finding. First, our sample is younger, mostly heterosexual, and overall has a lower HIV risk perception than the initial target population (i.e., sexual minority males) of adolescents and young adults identified for PrEP [6]. However, our sample is largely female which has important implications for HIV prevention among young racial/ethnic minority women. Increasing HIV incidence among racial/ethnic minority women and increasingly riskier sex practices among older aged adolescent girls suggest the need to continue to expand HIV prevention strategies, including PrEP, to female adolescents [27, 28]. Second, our qualitative findings reveal that PrEP stigma and the fear of being judged by family members and healthcare providers may affect adolescents’ willingness to use PrEP. Our findings are similar to other studies with adult samples in which negative beliefs about people who use PrEP (e.g., PrEP is for people who are promiscuous) and discomfort discussing PrEP with a provider were associated with disinterest in PrEP [29, 30]. These findings further support developing positive messaging about PrEP that targets adolescents, their families, and sociocultural barriers to PrEP access.

The finding that willingness to use PrEP is associated with older age and more education supports current recommendations to tailor PrEP promotion materials to older adolescents and highlights a need to increase access to PrEP information to adolescents with less education. Given the number of misconceptions adolescents report about PrEP, our study further underscores the need to ensure that PrEP information is culturally and developmentally appropriate for all sexually active adolescents. Similar to other studies with adult samples [12], we found that high risk behavior (i.e., condomless sex in the past six months) and higher risk perception of acquiring HIV are associated with willingness to use PrEP. These findings demonstrate the importance of developing PrEP implementation programs for Black and Latinx adolescents that address behavioral risk factors and include PrEP as part of comprehensive HIV prevention efforts. Lastly, qualitative discussions emphasized the role of healthcare providers in shaping adolescents’ beliefs about PrEP. These findings also signal the need for more investigations of the routes by which adolescents learn about PrEP (e.g., as part of routine primary care or within school-based health centers).

The results from this study should be considered in light of its limitations. All of the data collected in this study are based on self-report which may affect recall bias and social desirability. Nonetheless, the measures used in our survey to assess HIV risk and PrEP interests have been used in other studies [18–20]. Further, youth answered questions on a study tablet (community sample) or online which should address some concerns related to social desirability. We did not assess the specific type and amount of drugs or alcohol used during sex. This omission is a noted limitation as knowing the type and amount of drugs or alcohol used during sex could inform PrEP interventions for specific substance using profiles. Willingness to use PrEP was assessed using a hypothetical question. Although this question has been used in other studies, caution should be applied when interpreting our results for PrEP willingness as they may be overestimated. Due to the community-based sampling strategies used in this study, findings are not generalizable to all sexually active Black and Latinx adolescents in the U.S. For example, our qualitative data are gathered from a sample of adolescents in New York City and therefore may not be generalizable to adolescents in other regions in the U.S. Additionally, our findings are not generalizable to other high risk populations (i.e., gender queer, non-binary, and trans individuals) who are underrepresented in the study sample.

## Conclusions

This mixed-methods community-based study examined factors related to PrEP uptake among sexually-active Black and Latinx adolescents residing in U.S. cities with some of the highest burdens of HIV. Our findings show that HIV knowledge was low and risky sexual behavior was high within this sample which provides important insights into increasing PrEP uptake in this group. The majority of our sample were engaged in primary health care, which further supports the call to have PrEP discussed as part of routine primary care [31], especially for sexually active adolescents. Providing developmentally appropriate information during routine care has the potential to shift norms about adolescent sexual health and offset many of the challenges associated with curbing the spread of HIV among Black and Latinx adolescents and young adults including access to HIV prevention strategies, provider biases, and stigma.

## Supporting information

S1 Appendix Items from the qualitative guide and survey used in the analyses presented in the manuscript. (DOCX)
