# Supplementary material for: Awareness of and willingness to use PrEP among Black and Latinx adolescents residing in higher prevalence areas in the United States
Source: PLoS One. 2020 Jul 6;15(7):e0234821. doi: 10.1371/journal.pone.0234821 (PMC7337337; doi:10.1371/journal.pone.0234821)
Supplement: S1 Appendix — (DOCX) [file pone.0234821.s001.docx]

**Supporting Information**

Qualitative Items

Description: Below are the questions from the focus group and one-on-one interview guide analyzed for this manuscript

1) What have you heard about PrEP or the “HIV prevention pill”?

2) What are some of the concerns people your age might have about getting a prescription for PrEP from a doctor?

3) What are the chances that adolescents prescribed PrEP would take it daily?

4) Would you tell your parents that you are taking PrEP?;

a. What about your friends?

b. Boyfriend/girlfriend or sexual partner?

5) How do you think your parents would feel if you told them you wanted to take PrEP?.

a. What about your friends?

b. Boyfriend/girlfriend or sexual partner?

Quantitative Items

Description: Below are the questions from the survey analyzed for this manuscript

How old are you?

- 12 years and younger
- 13 years
- 14 years
- 15 years
- 16 years
- 17 years
- 18 years and older

Which of the following best describes your race/ethnicity?

- American Indian/Alaska Native
- Asian
- Black/African American
- Hispanic or Latino/a
- Native Hawaiian/Other Pacific Islander
- White
- Other race:_________________

What grade are you in/did you last complete)?

- - 6^th^ grade or lower
  - 7^th^ grade
  - 8^th^ grade
  - 9^th^ grade
  - 10^th^ grade
  - 11^th^ grade
  - 12^th^ grade
  - High school graduate or GED
  - Some college

How would you describe your gender?

- Female
- Male
- Transgender woman (biologically assigned male, but identify as a woman)
- Transgender man (biologically assigned female, but identify as a man)
- Gender queer
- Other

This question is about sexual orientation. Your sexual orientation might be different from your actual behavior. How would you describe your sexual orientation?

- Lesbian
- Gay
- Bisexual
- Heterosexual
- Queer
- Asexual
- Other
- I prefer not to say

Are you currently in a romantic relationship (a relationship that you consider your someone to be your girlfriend/boyfriend, main partner, or your girl/guy)?

- No
- Yes

In general, would you say you and your family have more money than you need, just enough for your needs, or not enough to meet your needs?

- - More than needed
  - Just enough
  - Not enough

What do you think the chances are that you will ever get HIV in your lifetime?

- - Not at all likely
  - A little bit likely
  - Somewhat likely
  - Very likely
  - Extremely likely

Have you ever been tested for HIV?

- Yes
- No
- I don’t know

Has a healthcare provider EVER in your life told you that you have… (Yes/No)

*Items:*

- Chlamydia
- Gonorrhea
- Syphilis
- Genital Herpes
- Hepatitis
- Bacterial Vaginosis (BV)
- Trichomoniasis (Trich)
- Human Papilloma Virus (HPV) or Genital Warts
- Other type of sexually transmitted infection______________

The following statements are about HIV, sexually transmitted infections (STIs), and pregnancy. Please indicate how true or false the following statements are:

Response Items:

1: Definitely False

2: Probably False

3: Don’t Know

4: Probably True

5: Definitely True

Questions:

- Birth control pills can protect a woman from sexually transmitted diseases including HIV.
- If a man pulls out right before orgasm, condoms don't need to be used to protect against the HIV.
- Most people who have a sexually transmitted infection (STI) or HIV look and feel healthy.
- A man is not likely to get HIV from having sex with a man unless he has had sex with other men (he is gay or bisexual).
- If you're seeing someone and if they agree not to have sex with other people, it is not important to use a condom.
- If a person has a sexually transmitted infection (STI) and doesn't get treated, eventually it goes away with no problems left.
- Vaseline and other oils should not be used to lubricate condoms.
- A pregnant woman with a sexually transmitted infection (STI) or HIV can pass it on to her baby during pregnancy or while giving birth.
- There is a cure for HIV and the government is keeping it from the public.
- HIV was created in a government laboratory.
- The government promotes the use of condoms in order to limit the number of births of certain groups.

Have you ever HEARD OF a daily pill that an HIV-negative person can take to prevent getting HIV? This pill is also called HIV pre-exposure prophylaxis, PrEP, and Truvada®.

- - No
  - Yes
  - I don’t know

How likely would you be to take PrEP (daily HIV prevention pill) if it were available for free?

- - Definitely would not take PrEP
  - Probably would not take PrEP
  - Might take PrEP
  - Probably would take PrEP
  - Definitely would take PrEP
